# Activity of FAAH-Inhibitor JZP327A in an Experimental Rat Model of Migraine

**DOI:** 10.3390/ijms241210102

**Published:** 2023-06-14

**Authors:** Rosaria Greco, Miriam Francavilla, Chiara Demartini, Anna Maria Zanaboni, Sara Facchetti, Michela Palmisani, Valentina Franco, Cristina Tassorelli

**Affiliations:** 1 Section of Translational Neurovascular Research, IRCCS Mondino Foundation, Via Mondino 2, 27100 Pavia, Italy; miriam.francavilla@mondino.it (M.F.); chiara.demartini@mondino.it (C.D.); annamaria.zanaboni@unipv.it (A.M.Z.); sara.facchetti@mondino.it (S.F.); michela.palmisani@mondino.it (M.P.); valentina.franco@unipv.it (V.F.); cristina.tassorelli@unipv.it (C.T.); 2 Department of Brain and Behavioral Sciences, University of Pavia, Via Bassi 21, 27100 Pavia, Italy; 3 Clinical and Experimental Pharmacology Unit, Department of Internal Medicine and Therapeutics, University of Pavia, Via Ferrata 9/A, 27100 Pavia, Italy

**Keywords:** migraine pain, trigeminal hyperalgesia, inflammation, endocannabinoid system

## Abstract

Increased anandamide levels via fatty acid amide hydrolase (FAAH) inhibition can decrease the pronociceptive responses and inflammatory mediators in animal models of migraine. Here, we profile the pharmacological activity of the FAAH inhibitor JZP327A, a chiral 1,3,4-oxadiazol-2(3H)-one compound, in the mediation of spontaneous and nocifensive behaviour in the animal models of migraine based on nitroglycerin (NTG) administration. JZP327A (0.5 mg/kg, i.p.) or vehicle was administered to male rats 3 h after NTG (10 mg/kg, i.p.) or NTG vehicle injection. The rats were then exposed to the open field test and an orofacial formalin test 1 h later. The levels of endocannabinoids and lipid-related substances, and the expression of pain and inflammatory mediators were evaluated in cranial tissues and serum. The findings show that JZP327A did not affect NTG-induced changes in the spontaneous behaviour of rats, while it inhibited NTG-induced hyperalgesia at the orofacial formalin test. Furthermore, JZP327A dramatically decreased the gene expression of calcitonin gene-related peptide (*CGRP*)*,* tumor necrosis factor alpha (*TNF-alpha*) and interleukin 6 (*IL-6*) in the trigeminal ganglia and medulla-pons, while it did not change endocannabinoids or lipids levels nor CGRP serum levels in the same tissues. These data suggest an anti-hyperalgesic role for JZP327A in the NTG model, which is mediated by the inhibition of the inflammatory cascade of events. This activity does not seem mediated by a change in the levels of endocannabinoids and lipid amides.

## 1. Introduction

There is evidence that endocannabinoid signalling imbalance may contribute to the pathophysiology of migraine [1]. Endocannabinoids (eCB) modulate calcitonin gene-related peptide (CGRP) release and dural mast cells activation, the main contributors to neurogenic inflammation [2]. The best-studied eCB, N-arachidonoylethanolamine (AEA, also known as anandamide) and 2-arachidonoylglycerol (2-AG), are produced in nociceptive regions, such as the epidermis, dorsal root ganglia, spinal cord, periaqueductal grey matter, and rostral ventromedial medulla [3]. 

AEA and lipid-related compounds, such as oleoyl ethanolamide (OEA) and palmitoylethanolamide (PEA), are degraded by the enzyme fatty acid amide hydrolase (FAAH), whereas 2-AG is degraded by the enzyme monoacylglycerol lipase (MAGL) [4]. Increased eCB signalling, achieved via catabolism inhibition, could be used to treat several disorders, including pain [5,6]. Indeed, numerous studies have found that FAAH inhibition promotes analgesia and reduces inflammation in animal models of acute inflammatory pain. Additionally, although the precise endocannabinoid-dependent processes causing migraine are unknown, the available evidence suggests that endocannabinoid system (ECS) activation could be a therapeutic strategy for lowering both the physiological and inflammatory components of pain that are involved in migraine attacks [1,7,8,9,10].

Several classes of selective FAAH inhibitors are based on oxazole, imidazole, and pyrazole; they have been described in the literature because of their crucial role in the hydrolysis of AEA [11]. Indeed, this branch of medicinal chemistry research on novel FAAH inhibitors is very promising [12]. Additionally, some of these drugs allowed for the demonstration that peripherally specific FAAH inhibition may be an effective strategy to treat pain, including migraine [13,14]. According to increasing evidence, the ECS appears to have a significant role in the processing and modulation of migraine pain [8,9,10,15]. In particular, both brain permeant (e.g., URB597) and impermeant (e.g., URB937) inhibitors of FAAH produce similar pharmacological effects in animal models of migraine on nociceptive tests, including the tail flick test as well as the orofacial and plantar formalin test [8].

The latest O-aryl carbamates [16,17] and the piperidine/piperazine ureas [18] are two classes of FAAH inhibitors that exhibited favourable activity and selectivity features in vivo. The former class includes N-cyclohexylcarbamic acid O-aryl ester derivatives, which feature a unique combination of anxiolytic, antidepressant and analgesic properties [15,19]. Thus, the discovery of novel FAAH inhibitors has taken centre stage in the pharmaceutical industry. Quantitative structure–activity relationship approaches aim to identify a meaningful relationship between the structure and the biological activity of certain groups of substances and involve the development of mathematical or computational models.

These techniques create a numerical connection between changes to chemical structure and those related to the bioactivity of 1,3,4-oxadiazol-2-one, one of the several scaffolds used to make FAAH inhibitors, and have lately gained attention in the production of serine hydrolase inhibitors, including FAAH [20,21]. 

There are currently other 1,3,4-oxadiazol-2(3H)-one compounds with strong affinity and good selectivity for FAAH; for instance, JZP327A was discovered to be a potent human recombinant FAAH inhibitor with an IC50 value of 11 nM and a >900-fold selectivity over MAGL and cyclooxygenase (COX) isoenzymes in vitro. The in vitro inhibitory actions of JZP327A are firmly bound and slowly reversible [20,21]. This compound showed high FAAH inhibitory selectivity and efficiency in vitro in areas involved in migraine aura, namely the cerebellum and cerebral cortex [22]. However, whether this compound is effective in vivo has not been tested so far. JZP327A would represent an alternative strategy to irreversible covalent inhibitors that are currently being investigated. Indeed, its properties would prevent the formation of covalent adducts with the FAAH enzyme, reducing the side effects. 

The current study aims at profiling in vivo the pharmacological activity of JZP327A in an animal model specific for migraine. Consequently, we have found anti-hyperalgesic effects of JZP327A, which are mediated by the inhibition of the inflammatory cascade.

## 2. Results

### 2.1. Open Field

**Figure 1:**
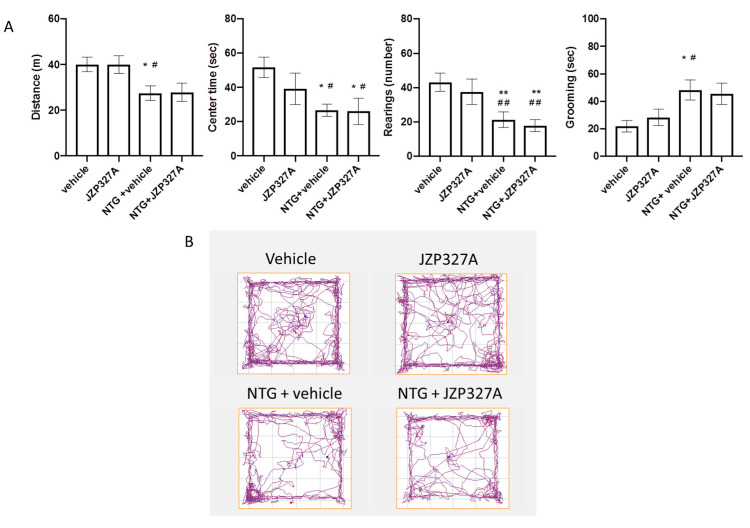
Open field test analysis. (**A**) Distance (expressed in meters) travelled in the apparatus; time spent (expressed in seconds) in the centre of the apparatus; number of rearing; time spent performing grooming behaviour (expressed in seconds). (**B**) Representative track plots of the different experimental groups. Male Sprague–Dawley rats were treated with JZP327A at a dose of 0.5 mg/kg, dissolved in a volume of 1 mL/kg of vehicle (tween-80/polyethylene glycol 200/saline) 3 h after NTG or vehicle injection. The locomotor activity, anxiety, and different types of behaviours were evaluated 4 h after NTG or vehicle injection. Data are expressed as mean ± SEM. One-way ANOVA followed by Tukey’s multiple comparisons test; * *p* < 0.05 and ** *p* < 0.01 vs. vehicle; # *p* < 0.05 and ## *p* < 0.01 vs. JZP327A. Vehicle: control group (n = 9); NTG + vehicle group: nitroglycerin + vehicle (n = 9); NTG + JZP327A group: nitroglycerin + JZP327A (n = 7); JZP327A group: nitroglycerin vehicle + JZP327A (n = 6).

The systemic administration of nitroglycerin (NTG) reduced the locomotor activity, expressed as distance travelled compared to the control (CT) group, confirming previous results [23]. Furthermore, it increased the anxiety-like behaviour as indicated by the reduced time spent in the centre of the open field and reduced the exploratory behaviour, expressed as the number of rearing, compared to the CT group (Figure 1). JZP327A did not change the locomotor activity or any other behaviour either when injected alone or with NTG, suggesting that it probably does not reach brain areas involved in modulating anxiety and depression.

### 2.2. Orofacial Formalin Test

**Figure 2:**
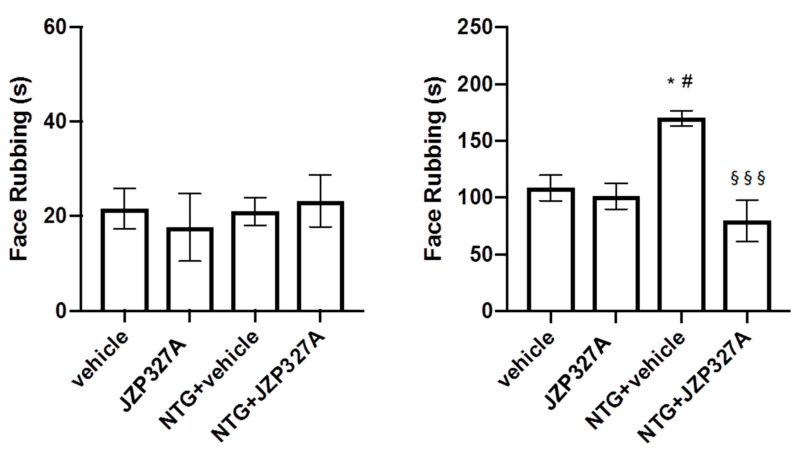
Orofacial formalin test. No significant differences between groups were seen during Phase I of the test. In Phase II, NTG administration (NTG + vehicle) significantly increased face rubbing behaviour compared to the vehicle group. JZP327A injected 3 h after NTG (NTG + JZP327A) significantly decreased face rubbing behaviour in Phase II compared to NTG + vehicle group. No changes were observed when JZP327A was administered alone (without NTG). Data are expressed as mean ± SEM. One-way ANOVA followed by Tukey’s multiple comparisons test: * *p* < 0.05 vs. vehicle; # *p* < 0.05 vs. JZP327A; §§§ *p* < 0.001 vs. NTG + vehicle. Vehicle: control group (n = 9); NTG + vehicle group: nitroglycerin + vehicle (n = 9); NTG + JZP327A group: nitroglycerin + JZP327A (n = 7); JZP327A group: nitroglycerin vehicle + JZP327A (n = 6).

In agreement with previous studies [15], NTG administration induced an increase in nocifensive behaviour (face rubbing) compared to the CT group in the second phase of the test, suggesting the presence of a hyperlagesic state (Figure 2).

JZP327A did not affect nocifensive behaviour when administered alone, while it significantly attenuated NTG-induced hyperalgesia when administered 3 h after NTG (NTG + JZP327A group). No significant difference among groups during Phase I of the test has been found. This finding reports a migraine-related anti-hyperalgesic effect of the inhibitor JZP327A.

### 2.3. Gene Expression

**Figure 3:**
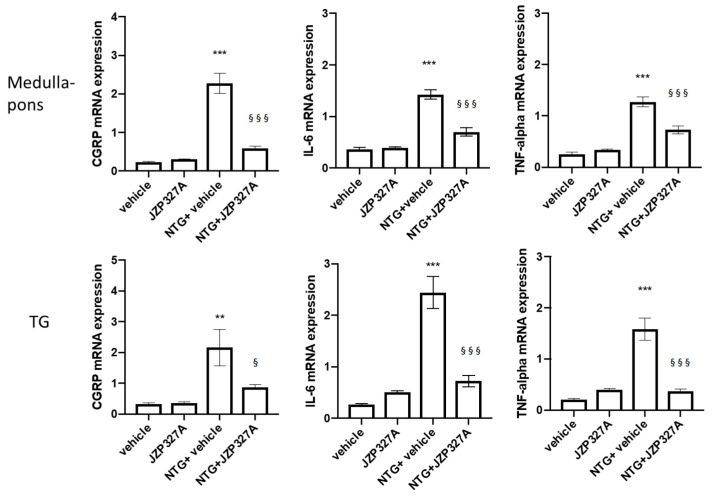
mRNA expression levels of *CGRP, IL-6* and *TNF-alpha* in the medulla-pons and trigeminal ganglion (TG). NTG treatment (NTG + vehicle group) significantly increased mRNA levels of all the genes evaluated compared to vehicle group in all areas. NTG-induced changes were prevented by the administration of JZP327A (NTG + JZP327A). No changes were found in the baseline conditions. Data are expressed as mean ± SEM. Tukey’s multiple comparisons test; ** *p* < 0.01 and *** *p* < 0.001 vs. vehicle; § *p* < 0.05 and §§§ *p* < 0.001 vs. NTG + vehicle. Vehicle: control group (n = 6); NTG + vehicle group: nitroglycerin + vehicle (n = 6); NTG + JZP327A group: nitroglycerin + JZP327A (n = 6); JZP327A group: nitroglycerin vehicle + JZP327A (n = 6).

In rats treated with NTG, the gene expression of tumour necrosis factor alpha (*TNF-alpha*), interleukin 6 (*IL-6*), and *CGRP* considerably increased in the medulla-pons and trigeminal ganglion (TG) (Figure 3). JZP327A prevented the NTG-induced increase in *CGRP*, *IL-6*, and *TNF-alpha* mRNA levels in these areas. When administered alone, JZP327A (without NTG) did not change gene expression in either of the evaluated areas. These data suggest the ability of JZP327A to counteract the NTG-induced activation of the inflammatory pathways.

### 2.4. Lipid Levels

**Figure 4:**
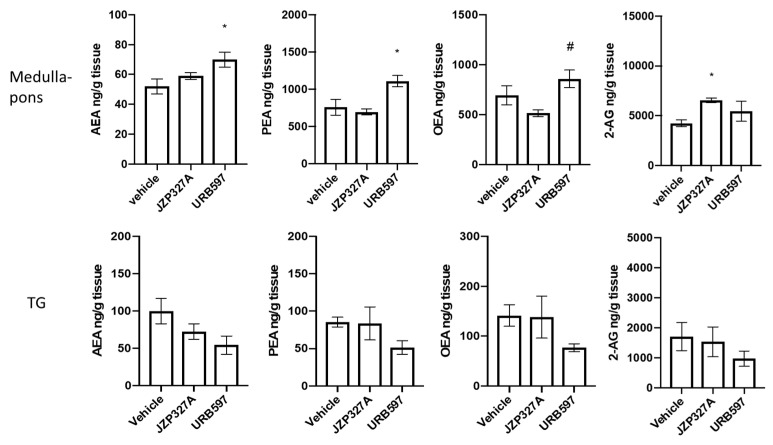
Lipid levels (expressed as ng/g tissue) in the medulla-pons and trigeminal ganglion (TG) of rats treated with vehicle, JZP327A (0.5 mg/kg, i.p.) or URB597 (2 mg/kg, i.p.). Data are expressed as mean ± SEM. Tukey’s multiple comparisons test; * *p* < 0.05 vs. vehicle; # *p* < 0.05 vs. JZP327A. Vehicle: control group (n = 6); JZP327A group (n = 6); URB597 group (n = 6).

**Figure 5:**
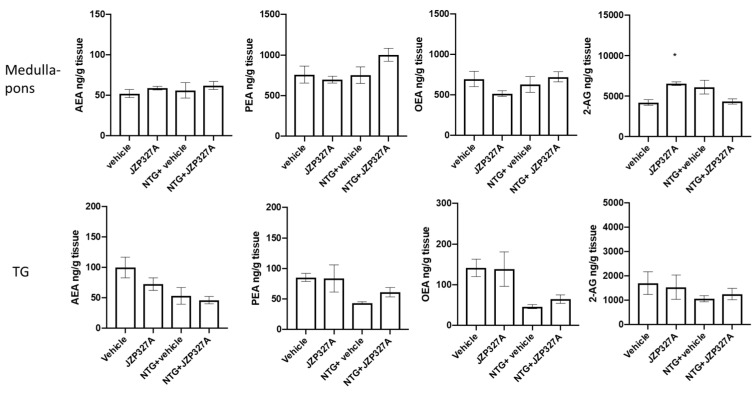
Lipid levels (expressed as ng/g tissue) in the medulla-pons and trigeminal ganglion (TG) of rats treated with NTG vehicle (vehicle), JZP327A, NTG + vehicle, and NTG + JZP327A (n = 6 per group). Data are expressed as mean ± SEM. Tukey’s multiple comparisons test; * *p* < 0.05 vs. vehicle.

JZP327A administration unexpectedly induced an increase in 2-AG levels in the medulla-pons (Figure 4), while it did not change the levels of the other evaluated lipids. URB597, on the other hand, increased AEA and PEA levels in the medulla-pons, whereas no effect was observed in TG, confirming previous results [8]. In the presence of NTG, either with JZP327A or its vehicle, no changes in lipid levels were reported in the medulla-pons and TG compared to vehicle (Figure 5). The results suggest that the JZP327A-related effects are probably not mediated by a change in specific levels of endocannabinoids and lipid amides.

### 2.5. CGRP Serum Level

**Figure 6:**
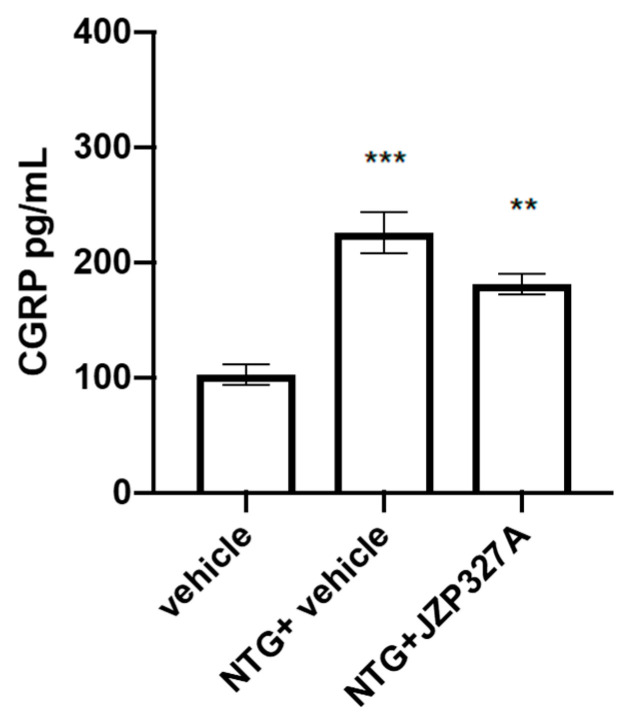
CGRP serum levels (pg/mL). Male Sprague–Dawley rats were treated with JZP327A at a dose of 0.5 mg/kg (i.p.) dissolved in a volume of 1 mL/kg of vehicle (tween-80/polyethylene glycol 200/saline) associated with NTG (10 mg/kg, i.p). Data are expressed as mean ± SEM. One-way ANOVA followed by Tukey’s multiple comparisons test; ** *p* < 0.01 and *** *p* < 0.001 vs. vehicle. Vehicle: control group (n = 6); NTG + vehicle group: nitroglycerin + vehicle (n = 6); NTG + JZP327A group: nitroglycerin + JZP327A (n = 6).

NTG treatment increased CGRP serum levels compared to the vehicle group. This change was not significantly affected by JZP327A administration (Figure 6). 

## 3. Discussion

FAAH inhibitors are effective in animal models of inflammation and pain, including migraine [7,8]. Most designed FAAH inhibitors are of the irreversible covalent variety. However, reversible FAAH inhibitors, as with OL-135, were also found to decrease mechanical allodynia in vivo in the rat spinal nerve ligature model [24]. Indeed, an irreversibly blocked enzyme would no longer exhibit its natural role of compensating for the endogenous accumulation of substrates. However, FAAH inhibitors should have the ability to achieve near-total enzymatic inhibition to efficiently produce analgesic effects in preclinical pain models. Based on in vitro enzymatic assays [20], JZP327A was chosen as the most intriguing derivative and was tested in vivo in this preliminary study. Previous work described a number of chiral 1,3,4-oxadiazol-2-ones, among which JZP327A was found to be a potent human recombinant FAAH inhibitor (IC50 = 11 nM) with >900-fold selectivity over MAGL and COX isoenzymes. JZP327A displayed a strong preference for FAAH in vitro since it showed no inhibition at 10 M against MAGL, endocannabinoid alpha/beta-hydrolase domain (ABHD) 6 and ABHD12, the other brain serine hydrolases. The in vitro selectivity and potency of JZP327A for FAAH inhibition were confirmed in the cortical areas of rats [22]. In the present in vivo investigation, JZP327A counteracted NTG-induced trigeminal hyperalgesia and the activation of pain and inflammatory pathways (CGRP, TNF-alpha, and IL-6) in the TG and medulla-pons, without affecting NTG-related hypomotility and anxiety behaviours [15] as well as an NTG-induced increase in CGRP serum levels. The differential effect of JZP327A on NTG-induced changes in *CGRP* mRNA expression (which was prevented in the nervous tissues) and in CGRP serum levels (which were unchanged) was unexpected, and it is not easy to explain. It is possible to speculate that the administration of the compound 3 h after NTG could not intercept the mobilization of CGRP from the trigeminovascular terminals induced by NTG, while it did block the synthesis of new CGRP in the soma of neurons located in the medulla-pons and in the TG [25,26,27,28,29]. Another possible explanation is that the effect of JZP327A on CGRP mRNA expression is due to feedback inhibition of the inflammatory pathway, whereas it is not able to act on the CGRP release machinery [30].

In the experimental setting adopted in this study, NTG did not induce any change in the eCB and lipid levels in both tissues under investigation and the treatment with JZP327A did not alter eCB levels, contrary to what we have observed previously with the peripherally restricted FAAH inhibitor URB937 [13]. This suggests that JZP327A’s activity on the orofacial formalin test takes place via a non-FAAH-mediated suppression of proinflammatory responses and *CGRP* gene expression, as we reported in earlier research with other inhibitors [10,15]. It is probable that at the dose tested in the present study, JZP327A may inhibit 2-AG oxygenation and contribute to the reduction in several eicosanoids, including prostaglandin (PG) E2, PGD2, PGF2α, and thromboxane B2 [31], thus reducing pain and inflammation. Alternatively, we can speculate that nitric oxide released from NTG may trigger the formation of metabolites produced by JZP327A, which may induce the observed anti-hyperalgesic effect that is directly unrelated to increased endocannabinoid levels in the medulla or other cranial areas [32]. A similar pattern has been reported for phenylmethylsulfonyl fluoride, a serine protease inhibitor at a specific dosage, that, at higher doses, may induce biological effects unrelated to the ECS [14]. Other mechanisms and mediators are also possible, given that eCB components interact with an extended set of non-cannabinoid mediators and feature pleiotropic metabolizing enzymes in central and peripheral nervous system areas, making it difficult to target specific molecular mechanisms [14,33,34]. It is important to note that the eCB levels here were measured in two discrete cranial regions, not in the entire brain, as reported for other inhibitors by most studies in the literature.

The open field test revealed no appreciable impact on spontaneous exploratory behaviour and motor activity, confirming the effects of other FAAH inhibitors [35].

In the absence of NTG, we observed a dissociation between in vitro and in vivo effect of JZP327A; JZP327A inhibits FAAH activity in vitro, but it proved ineffective in vivo in this study. Unexpectedly, we detected increased 2-AG levels in the medulla pons (but not in TG), suggesting the possibility that JZP327A may have a higher affinity for enzymes involved in 2-AG catabolic pathway in vivo. This increase was not associated with significant changes in the orofacial formalin test or mRNA levels. It is worth noting that the observed effect on 2-AG was borderline statistically significant compared with the control group.

### Limitations of the Study

This study shows the performance in vivo of JZP327A in an animal model of migraine for the first time, but the interpretation of the results should be performed with caution due to some limitations. First, we tested only one dose of JZP327A, and we cannot rule out the possibility that higher or lower doses might involve eCB signalling. A dose–response study is necessary to obtain definite evidence for this possibility. In the absence of data about the activity of JPZ327A in vivo, in this first study, we selected the dose of 0.5 mg/kg as it seemed the best compromise when considering the high FAAH inhibitory potency of JPZ327A in vitro [20] and the need to avoid deleterious effects on motor function or catalepsy. However, it cannot be excluded that an even lower dose of JZP327A may cause an increase in AEA levels in the cranial areas. Indeed, there is evidence that the metabolism pathway is relevant in vivo under certain conditions and that multiple enzymes may be involved in the metabolism of ECS [36]. These catabolic pathways represent an interesting point of intersection between endocannabinoid and classical eicosanoid systems, leading to the production of new biologically active metabolites [36]. For instance, cytochromes P450 (CYPs) are known to epoxidize lipids into anti-inflammatory and antipain mediators that are more effective than the parent molecules. In agreement, the CYP-mediated metabolism of eCBs was recently shown to produce epoxy-eCBs that exhibit CB2 receptor selectivity and are anti-inflammatory [37].

Second, we only tested the effect of JZP327A when administered 3 h after NTG, and we lack knowledge about its possible activity when administered closer to or before NTG. Additional studies also in female rats are required to address these limitations, and to confirm or refute and integrate the present data.

## 4. Materials and Methods

### 4.1. Animals

Male Sprague Dawley rats (150–175 g, Charles River Laboratories, Calco, Italy) were housed in pairs in cages at the University of Pavia’s animal facility under carefully monitored conditions: temperature 21–22 °C, relative humidity 60–50%, and 12/12 h light cycle, with water and food ad libitum. The Italian Ministry of Health (376/2020-PR) approved all procedures, which were performed in compliance with the guidelines of European Community Directive 2010/63/EU of 22 September 2010. Before the experimental testing, animals were given a week to become familiarised with their housing conditions after arrival.

### 4.2. Drugs

NTG (Bioindustria L.I.M., Novi Ligure, Italy) was prepared from a stock solution of 5.0 mg/1.5 mL dissolved in 27% alcohol and 73% propylene glycol. The NTG solution was diluted in saline (0.9% NaCl) to reach a final concentration of alcohol of about 6% and propylene glycol of 16% and was administered intraperitoneally (i.p.) at a dose of 10 mg/kg. The NTG vehicle contained saline, alcohol 6%, and propylene glycol 16%. 

**Figure 7:**
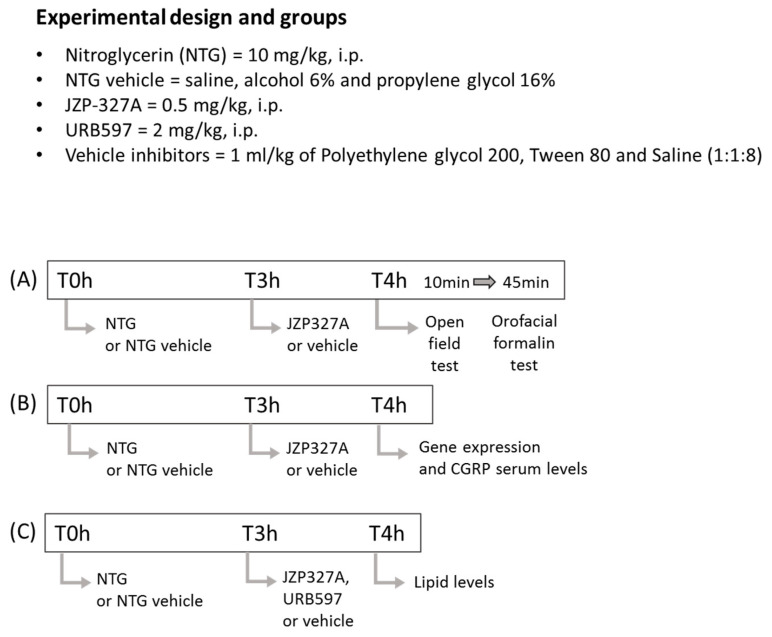
Experimental timeline for the treatment and testing procedures in the three experimental settings used in this study. (**A**) JZP327A (or vehicle) was administered 3 h after NTG (or its vehicle), meaning 1 h before the open field test; immediately after the open field, rats were injected with formalin (50 μL, s.c.) and the orofacial formalin test was performed (45 min duration). (**B**) Rats were immediately sacrificed after the drug treatment schedule for the detection of gene expression in cranial tissues and the assay of CGRP serum levels. (**C**) JZP327A or URB597 (or vehicle) was administered 3 h after NTG (or its vehicle); rats were immediately sacrificed after the drug treatment schedule for lipid assays.

JZP327A (synthetized by the Department of Chemistry, University of Eastern Finland) was delivered systemically (i.p.) at a dose of 0.5 mg/kg in a volume of 2 mL/kg after being dissolved in tween-80/polyethylene glycol 200/saline (10/10/80; utilized as a vehicle) 3 h after NTG or vehicle. One hour later, a first set of rats underwent the open field test (10 min duration) and immediately after the open field test, they were injected with formalin (50 μL, s.c.) in the upper lip to perform the orofacial formalin test (45 min duration) (Figure 7A) [15]. At the end of the orofacial formalin test, rats were euthanized under deep anaesthesia (sodium thiopental). 

In order to assess the gene expression of *CGRP*, pro-inflammatory cytokines, AEA, PEA and OEA levels (see Section 4.6) in the medulla-pons (bregma, 13.30 to 14.60 mm) and TG, a second set of rats, undergoing the same treatment schedule, was sacrificed to collect the regions of interest. We also assayed serum levels of CGRP (Figure 7B). URB597 (3′-carbamoyl-biphenyl-3-yl-cyclohexylcarbamate, Cayman Chemical, Ann Arbor, MI, USA) was dissolved in tween-80/polyethylene glycol 200/saline solution (10/10/80; used as vehicle) and injected i.p. at a dose of 2 mg/kg 3 h after NTG (or vehicle). The medulla-pons and TG were also collected from URB597-treated rats, following the same timeline scheduled for JZP327A, as a positive control for JZP327A only for lipid assay (Figure 7C).

### 4.3. Open Field Test

The open field test allows for the evaluation of locomotor activity, anxiety, and several types of behaviours, such as rearing and grooming. The open-field arena consisted of a 92 cm × 92 cm box (Ugo Basile, Gemonio, VA, Italy), with grey opaque walls that fit solidly into a grey nonreflective base plate. Each rat was acclimatised to the test room for 60 min before testing. One minute before testing, each rat was placed into a cylinder chamber in the centre of the arena for 1 min; the aim of this was to reduce the locomotion resulting from experimenter handling [15]. After removing the cylinder, the rat was allowed to freely explore the arena and was recorded for 10 min using a video camera placed over the arena. The test arena was cleaned prior to use with 70% ethanol after each test. The measurement of the distance travelled in the entire arena, the time spent in the centre and rearing behaviour were used to assess locomotor activity, anxiety, and exploration, respectively [23]. Additionally, as a sign of heightened nociception, we assessed spontaneous grooming behaviour. All parameters were completed by a trained observer who was blinded to the treatment, using the ANY-Maze software (Ugo Basile, application version 4.99 g Beta). The open field arena was divided into 16 square units using the ANY-Maze software, with 4 squares serving as the centre and 12 squares along the outer perimeter serving as the periphery.

### 4.4. Orofacial Formalin Test

Rats were acclimatized to the test chamber in the days before the orofacial formalin test for 10 min. On the experimental day, they were injected subcutaneously with 50 μL of formalin 1.5% (*v*/*v*) into the right upper lip. Face rubbing was evaluated by a researcher blind to treatments, counting the seconds the animal spent grooming the injected area with the ipsilateral forepaw or hind paw 0–3 min (Phase I) and 12–45 min (Phase II) after formalin injection.

The observation box was a 30 cm × 30 cm × 30 cm glass chamber with mirrored sides. A camera, recording face rubbing time for offline analysis, was located at a distance of 50 cm from the box to provide a clear view of each rat.

### 4.5. Gene Expression

Four hours after receiving NTG or the vehicle injection, the rats were sacrificed under deep anaesthesia (sodium thiopental). After decapitation, the medulla pons (bregma, −13.30 to −14.60 mm) and TG were dissected out, rinsed in sterile 0.9% NaCl cold solution, placed in cryogenic tubes and kept at −80 °C until rt-PCR processing for *CGRP*, *TNF-alpha*, and *IL-6* gene expression. 

The procedures were carried out in an RNase-free environment; following RNA extraction, all RNA samples had absorbance ratios (260/280 nm) that varied from 1.9 to 2.0, indicating no significant protein contamination, including that of blood origin.

Primer sequences, obtained from the AutoPrime software (http://www.autoprime.de/AutoPrimeWeb; accessed on 26 April 2018) are reported in Table 1. Glyceraldehyde 3-phosphate dehydrogenase (*GAPDH*), whose expression remained constant in all experimental groups, was used for normalization. All samples were assayed in triplicate, and the 2^−∆∆Ct^ = 2^−(∆Ct gene−∆Ct housekeeping gene)^ method was used to investigate the differences in gene expression levels.

### 4.6. AEA, 2-AG, PEA and OEA Assay 

The Medulla pons and TG were weighed before homogenization in methanol containing cannabidiol-d3 as an internal standard. Three volumes of the internal standard solution (500 ng/mL in methanol with 0.8% formic acid) and nine volumes of methanol containing 0.8% formic acid were added to each sample according to the weight of each rain tissue section. After 10 min of centrifugation (17,000× *g* at 4 °C), the supernatant was transferred to an autosampler vial and injected into an online solid-phase extraction liquid chromatography–electrospray tandem mass spectrometry setup according to the procedure of Fanelli et al., following refinement [38].

Briefly, samples were analysed using a 3200 QTRAP^®^ triple quadrupole mass spectrometer (Applied Biosystems Sciex, Darmstadt, Germany) used in conjunction with a high-pressure liquid chromatography (HPLC) ExionLC 100 integrated system, equipped with a quaternary low-pressure mixing pump, an autosampler, a column oven, a degasser and a controller (Applied Biosystems Sciex, Darmstadt, Germany). The chromatographic column was a monolithic C18 column (Onyx, 100 mm × 3 mm i.d., Phenomenex, Bologna, Italy) maintained at 25 °C. Gradient elution mobile phases consisted of A (water/methanol 98:2 *v*/*v* containing 10 mM ammonium formate and 0.1% formic acid) and B (methanol/ acetonitrile/isopropanol 80:10:10 *v*/*v* containing 8 mM ammonium formate and 0.08% formic acid). 

A solid phase extraction technique was employed online using a perfusion column (POROS R1, 30 mm × 2.1 mm inner diameter, 20 μm particle size, Thermo Fisher Scientific, Waltham, MA, USA). To initiate the process, a 50 µL portion of the sample was moved from the HPLC system to the perfusion column, which was inserted into the loading position of a 10-port valve starting with a mobile phase consisting of 94.6% A and 5.4% B, flowing at a rate of 1.5 mL/min for one minute. After that, the valve was switched to the injection position, and the lipids were transferred to the analytical column where their separation was achieved at a flow rate of 0.5 mL/min through the following gradient: 90% A and 10% B (from 1 to 3.20 min); 14% A and 86% B (3.20 to 5.20 min) maintained up to 8.50 min; 11% A and 89% B (8.50 min to 9 min); and 8% A and 92% B (9 min to 12 min). Following this, a 5 min cleaning phase using a 1% B: 99% C mixture (the mobile phase C being pure methanol) at 0.6 mL/min was performed. During the last 3 minutes of the run, the perfusion column was re-equilibrated for 3 min at 0.6 mL/min to the initial conditions (94.6% A, 5.4% B). The total duration of the chromatographic run was 20 min.

### 4.7. CGRP Serum Level

Rats were sacrificed with a lethal dose of anaesthetics followed by decapitation. Truncal blood was centrifuged for 15 min at 1000× *g* at 2–8 °C for CGRP serum evaluations. CGRP levels were measured using commercial ELISA kits (α-CGRP: Elabsciences, Houston, TX, USA). The samples’ measured absorbance was compared to a standard curve using a microplate reader (Biotek, Santa Clara, CA, USA).

### 4.8. Statistical Analysis 

To determine the minimal sample size needed to achieve the experiments, we considered the nociceptive response in Phase II of the orofacial formalin test (face rubbing time) as the primary outcome. An a priori power analysis was conducted to obtain a statistical power of 0.80 at an alpha level of 0.05 (GPower 3.1). Based on a previous study [13], we hypothesized a difference in total face rubbing time between NTG-treated rats (mean 180 ± 36) and those injected with NTG + JZP327A of about 70 s (mean 109 ± 48), and thus, we estimated a sample size of 6 rats in each experimental group with an effect size of 1.68. However, due to the intergroup variability seen in the orofacial formalin test, we used a maximum of 9 rats per group for this test. All data were tested for normality using the Kolmogorov–Smirnov (K-S) normality test and considered normal. Differences among groups were compared by one-way ANOVA followed by post hoc Tukey’s multiple comparisons test. Statistical significance was set at *p* < 0.05. All statistical analyses were performed using GraphPad Prism software (version 8). Data were expressed as mean ± SEM.

## 5. Conclusions

We report here for the first time the anti-hyperalgesic and anti-inflammatory activity of JZP327A in an animal model of migraine. These preliminary findings suggest a therapeutic potential of this compound in migraine pain. However, a dose–response study with a time course is needed to investigate the effect of JZP327A on FAAH and MAGL activities and lipid levels in ex vivo preparations.

## Figures and Tables

**Table 1:** Sequences of primers used for rt-PCR.

Gene	Forward Primer	Reverse Primer
*GAPDH*	AACCTGCCAAGTATGATGAC	GGAGTTGCTGTTGAAGTCA
*CGRP alpha* (*CGRP*)	CAGTCTCAGCTCCAAGTCATC	TTCCAAGGTTGACCTCAAAG
*IL-6*	TTCTCTCCGCAAGAGACTTC	GGTCTGTTGTGGGTGGTATC
*TNF-alpha*	CCTCACACTCAGATCATCTTCTC	CGCTTGGTGGTTTGCTAC

## Data Availability

The data presented in this study are available from the ZENODO repository https://doi.org/10.5281/zenodo.7908712.
